# A Visuoperceptual Measure for Videofluoroscopic Swallow Studies (VMV): A Pilot Study of Validity and Reliability in Adults with Dysphagia

**DOI:** 10.3390/jcm11030724

**Published:** 2022-01-29

**Authors:** Katina Swan, Renée Speyer, Martina Scharitzer, Daniele Farneti, Ted Brown, Reinie Cordier

**Affiliations:** 1 Curtin School of Allied Health, Faculty of Health Sciences, Curtin University, Bentley, WA 6102, Australia; katina.swan@postgrad.curtin.edu.au (K.S.); renee.speijer@isp.uio.no (R.S.); 2 Department Special Needs Education, University of Oslo, 0315 Oslo, Norway; 3 Department of Otorhinolaryngology and Head and Neck Surgery, Leiden University Medical Centre, 2333 ZA Leiden, The Netherlands; 4 Department of Biomedical Imaging and Image-Guided Therapy, Medical University of Vienna, Waehringer Guertel 18-20, 1090 Vienna, Austria; martina.scharitzer@meduniwien.ac.at; 5 Audiologic Phoniatric Service, Infermi Hospital Rimni, 47900 Rimini, Italy; lele.doc@libero.it; 6 Department of Occupational Therapy, Faculty of Medicine, Nursing and Health Sciences, Monash University—Peninsula Campus, Frankston, VIC 3199, Australia; ted.brown@monash.edu; 7 Department of Social Work, Education and Community Wellbeing, Northumbria University, Newcastle upon Tyne NE7 7YT, UK

**Keywords:** classic test theory, dysphagia, measure, psychometrics, videofluoroscopic swallow studies, VMV

## Abstract

The visuoperceptual measure for videofluoroscopic swallow studies (VMV) is a new measure for analysing the recordings from videofluoroscopic swallow studies (VFSS). This study evaluated the reliability and validity of the pilot version of the VMV using classical test theory (CTT) analysis, informed by the consensus-based standards for the selection of health measurement instruments (COSMIN) guidelines. Forty participants, diagnosed with oropharyngeal dysphagia by fibreoptic endoscopic evaluation of swallowing, were recruited. The VFSS and administration of bolus textures and volumes were conducted according to a standardised protocol. Recordings of the VFSS were rated by three blinded raters: a speech-language pathologist, a radiologist and a phoniatrician. Inter- and intra-rater reliability was assessed with a weighted kappa and resulted in 0.889 and 0.944 overall, respectively. Structural validity was determined using exploratory factor analyses, which found four and five factor solutions. Internal consistency was evaluated with Cronbach’s alpha coefficients, which found all but one factor scoring within an acceptable range (>0.70 and <0.95). Hypothesis testing for construct validity found the expected correlations between the severity of dysphagia and the VMV’s performance, and found no impact of gender on measure performance. These results suggest that the VMV has potential as a reliable and valid measure for VFSS. Further validation with a larger sample is required, and validation using an item response theory paradigm approach is recommended.

## 1. Introduction

Oropharyngeal dysphagia (OD) is a disorder that disturbs the sensory and physical processes of swallowing [1]. As not all aspects of OD can be observed externally, investigation of OD often necessitates the use of specialised instrumental examination procedures. The videofluoroscopic swallow study (VFSS) is an instrumental exam that uses recordings of dynamic fluoroscopies in an assessment of swallowing physiology and kinematics. VFSS is recognised as a gold-standard instrumental swallowing assessment and is widely used in clinical and research settings around the world [2]. However, the video recordings require skilled analysis for meaningful interpretation. Clinicians typically examine the videos by visuoperceptual means to make judgments about impairments and to plan and trial interventions [3]. Measures suitable for visuoperceptual analysis of dynamic images with robust psychometric properties are therefore essential for the assessment and treatment of OD.

Several visuoperceptual analysis measures have been developed for VFSS. Some target a single construct, such as aspiration, while others attempt to measure multiple constructs, such as lingual and pharyngeal movement, residue, cough and upper oesophageal sphincter (UES) function [4]. The constructs included in measures are just one facet the clinician must consider when choosing an appropriate tool for OD analysis. Measures must be reliable, valid and responsive, with key psychometric properties that describe whether a measure evaluates what it claims to assess and whether it does so in a consistent, repeatable manner [5].

Understanding the psychometric properties of OD measures is important given the complexity of OD as a clinical and diagnostic construct, where a phenomenon viewed on VFSS may be interpreted in multiple ways. For example, the presence of pharyngeal residue may be explained by any of the following: the contrast material preparation in the oral phase (weak tongue squeeze), poor pharyngeal constriction, anatomical abnormalities, surgeries obstructing bolus flow, impaired upper oesophageal sphincter functioning, and other dysfunctions [6]. Analysis of psychometric properties provides statistical evidence about the relationships between the items in the measure, the precision of the scale, and the association between the measure and the construct(s) of interest [7].

In recent years, the science of psychometric analyses applied to outcome measures has been scrutinised through the consensus-based standards for the selection of health measurement instruments (COSMIN) initiative [8]. The COSMIN initiative applied international multi-disciplinary expertise in psychometrics, research and measure development to formulate a methodology for evaluating outcome measures [5]. In a series of Delphi studies, consensus was reached on standardised definitions of psychometric properties, quality criteria for which properties should be reported, and recommended statistical methods to be used to investigate them. The COSMIN taxonomy encompasses nine psychometric properties, divided into three domains: reliability, validity and responsiveness [9,10,11,12,13]. The COSMIN checklist is an inventory of recommended criteria and statistical methods for studies on measurement properties [8].

The checklist was applied to VFSS visuoperceptual measures in a 2018 study, where psychometric properties were assessed in a combination of COSMIN ratings and quality criteria [4]. The authors found that visuoperceptual VFSS measures had overall indeterminate, limited or conflicting evidence of psychometric quality and concluded that there was insufficient evidence to recommend any of the VFSS measures reviewed [4]. Unclear or inadequate psychometric properties risk misapplication of the measure, while inaccurate measurement wastes resources and undermines the evidence base for clinical practice [14]. Thus, there is an urgent need for studies that focus on the development of VFSS measures that utilise sound statistical methods.

A new measure, the visuoperceptual measure for videofluoroscopic swallow studies (VMV) was created to address this gap. The process of developing a measure involves conceptualisation of the construct of interest, item/response scale generation (content validity), piloting the measure, preliminary evaluation, item refinement and reduction, and finally a large trial [15]. The VMV’s content validity was established in an international Delphi study involving more than 50 experts in OD and VFSS from 27 countries. The constructs to be included in the VFSS analysis, the conversion of these constructs to items, and the operationalisation of these items were established via consensus across three Delphi rounds. The Delphi identified 32 constructs recommended for analysis, and between one and four items per construct [16]. These findings were used to create the pilot version of the VMV, which comprised 97 items. As a new measure, its psychometric properties are not established. Therefore, the aim of this study is to conduct a pilot evaluation of the VMV’s psychometric quality. Specifically, the objectives are to evaluate the following psychometric properties:

- inter- and intra-rater reliability
- structural validity
- internal consistency
- hypothesis testing for construct validity

## 2. Methods

### 2.1. Participants

This study was granted ethical approval by the Human Research Ethics Committees of The Medical University of Vienna And Curtin University (HRE2018-0151, April 2018 and March 2019). Adults with OD, diagnosed by fibreoptic endoscopic evaluation of swallowing (FEES) and referred for VFSS as part of their assessment plan, were recruited from the Medical University of Vienna between July 2019 and March 2020. As FEES and VFSS are complementary instrumental assessments, diagnosis of OD by FEES supported appropriate participant selection [17]. Informed consent was obtained from all participants. 

In- and out-patients accessing services for OD were eligible if they satisfied the following inclusion criteria: (1) adults (>18yo) with a diagnosis of OD, (2) had been deemed by their treating clinician to be medically and cognitively appropriate for VFSS, and (3) to require VFSS to assess or manage their OD. Participants who had radical surgery of the head and/or neck were excluded.

A total of 40 participants were recruited. One patient was excluded due to data loss on the medical archive imaging system. Of the 39 remaining, 64% (*n* = 25) of participants were men and 36% (*n* = 14) were women. Participants ranged in age from 21 to 91 years (mean = 63.0 years, SD ± 17.0 years). The onset of OD (defined as the date of first symptoms) ranged from six days to five years prior to the VFSS, with onset less than 1 month prior to VFSS for 33% (*n* = 13), 1–6 months prior for 36% (*n* = 14), 7–12 months prior for 13% (*n* = 5), and more than 13 months prior for 18% (*n* = 7). Medical diagnoses in the participant group, while heterogeneous, can be grouped into four categories: cancer (primarily of head and neck), neurological disorder, surgery, and anatomical abnormality (Table S1—Aetiology of Oropharyngeal Dysphagia). Participants were assigned to groups based on the diagnosis which appeared most strongly associated with their OD diagnosis, based on consensus of the three authors (KS, RS, RC). For example, a participant with a history of lung cancer 20 years prior to VFSS who had a stroke the month before the VFSS was classified as ‘Neurological disorder’. The anatomical abnormality group was comprised of conditions in which physical changes to bodily structures adjacent to and involved in the process of swallowing were the most likely cause of the OD (e.g., cervical spine abnormalities).

### 2.2. Equipment and Materials

The VFSS were performed by a radiologist on a fluoroscopy unit (Axiom Sireskop S3 fluoroscopy system, Siemens Healthineers; Siemens AG, Erlangen, Germany) with a Siricon high-dynamic-range image intensifier, spot film device and analog/digital acquisition at an image rate of 30 pulses/s. Patients were placed upright in a sitting position. The oropharynx and the proximal oesophagus were viewed in lateral projection and anterior–posterior positions. Boluses consumed was comprised of a non-ionic low-osmolar contrast (Omnipaque™), thickener (Nutilis Clear^®^), water and a cracker (Mini Toast, Delhaize^®^; Delhaize Group SA, Brussels, Belgium). 

### 2.3. Protocol

Participants had part of their VFSS conducted according to a standardised protocol (Supplementary Materials, Figure S1). The protocol was designed to ensure participant safety by starting with small volumes of each texture (5 mL) and including cessation points if severe aspiration or residue was observed by the radiologist. As swallowing is affected by volume, texture and verbal instructions [18,19], the protocol included four different textures. These were administered using a standardised order, method and set of verbal instructions to maximise the variety of swallowing behaviours related to textures/volumes elicited, while controlling for the influence of the administrator. Textures/volumes were as follows: three trials of Thick (L3 International Dysphagia Diet Standardisation Initiative (IDDSI)), three trials of Thin (L0 IDDSI), four trials of pudding (L4 IDDSI), and one cracker (L7 IDDSI) [20] in four different volumes (5 mL, 10 mL, 20 mL, bite sized cracker). The average number of trials completed by the participants was seven (range 1–11), with the most common trial completed being 5 mL thick (completed by all 39 participants; however, data were lost from one due to technical issues, resulting in data from 38 participants for this trial). The VFSS was conducted by an experienced radiologist (>10 years’ experience). The non-ionic low-osmolality contrast was mixed with food and fluids according to a standard recipe (Supplementary Materials, Table S2: Administration protocol [21]) and each was kept at room temperature prior to the procedure.

In addition to the VFSS, assessment of the participants’ self-reported and observed functional health status (FHS) and clinician-perceived symptoms on VFSS were completed. Measures of FHS assess severity of OD symptoms from the perspective of daily functioning and impacts on participation in daily activities [22]. Participants who were referred for VFSS completed the Deglutition Handicap Index, Symptom subscale (DHI-S) [23] (a self-report FHS measure), and clinicians scored the Functional Oral Intake Scale (FOIS) [24] (an observational FHS measure) along with a 5-point ordinal scale to indicate the radiologist’s overall impression of OD severity based on viewing VFSS. To overcome the pragmatic limitation of having a single rater scoring OD severity, data were triangulated by using two separate measures of OD severity. (Supplementary Materials, Table S3. Functional Health Status and Severity measures and Table S4. Functional Health Status and Severity measures scoring.).

### 2.4. Manual

A manual was constructed based on Delphi study results. This manual includes: detailed instructions on contrast preparation, administration, patient positioning, items with descriptions, response scales, instructions for rating items, and anchor images [16].

### 2.5. Raters, Consensus Meetings, Training and Rating

**Figure 1:**
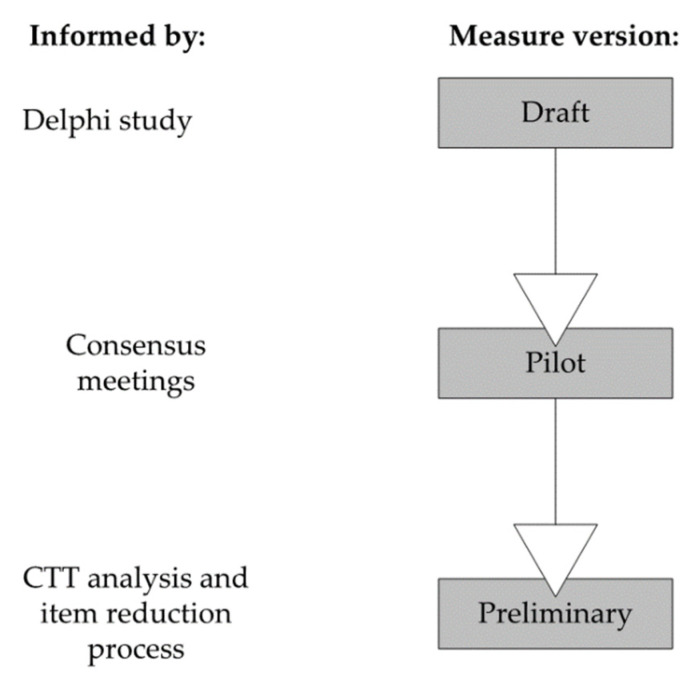
Overview of measure development and versions of the VMV.

Three raters used the draft VMV, informed by the Delphi results [16]. One rater had qualifications in speech-language pathology, the others were physicians with qualifications in radiology and phoniatrics, respectively. All three raters had over 10 years of experience with OD and VFSS. In a consensus meeting, the raters scored one patient through a full protocol, including all 11 trials, working item by item as a group. Each draft VMV item was discussed, and the manual regularly referenced by the raters on the first trial (5 mL Thick). As the raters progressed through the trials in the protocol, only new items were discussed in detail unless there was disagreement in scoring. Adjustments were made to items and the manual based on this feedback. These adjustments included removing ambiguous language, adding additional anchor images and expanding response options. After six hours, a 100% group consensus was reached for each item. The raters then scored three VFSS recordings independently and convened for an additional two-hour consensus meeting to discuss questions about measure use and resolve any differences in ratings. This consensus process led to the development of the pilot version of the VMV. An overview of measure development and versions of the VMV is depicted in Figure 1.

All of the VFSS recordings were deidentified. Ratings were completed on 100% of recordings using the pilot VMV on Qualtrics (www.qualtrics.com accessed on 3 July 2020). The raters referred to the manual as needed. At least two weeks after initial rating, repeated ratings were completed on an additional six (15%) randomly selected participants’ recordings by all three raters.

### 2.6. Item Reduction

The pilot version of the VMV included 97 items, derived from results of the international Delphi study on visuoperceptual analysis of VFSS [16] and informed by the results of the consensus meetings regarding the draft version. After completing the ratings, the raters and the authors met to review the pilot version of the VMV item by item and reach consensus on whether each item should be kept, modified or rejected in the next iteration of the measure.

**Figure 2:**
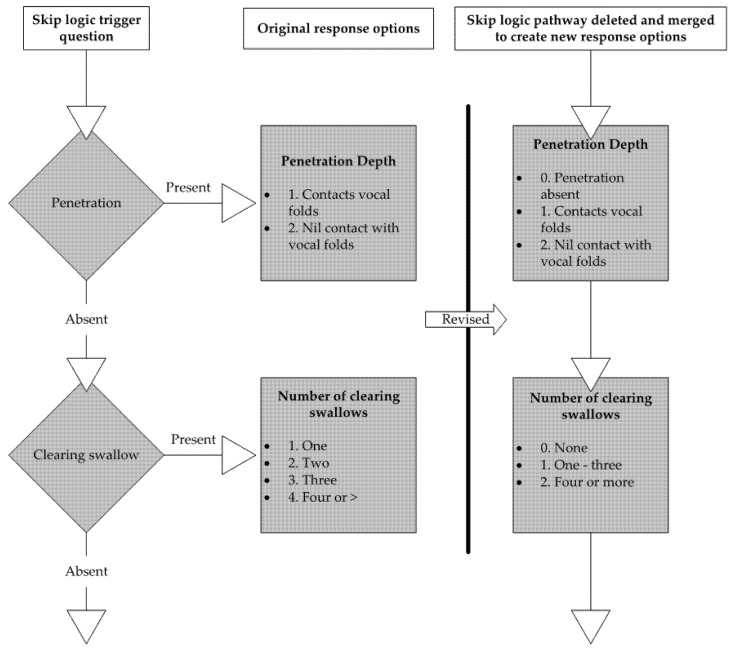
Skip logic—original question structure vs. removed skip logic with retained concept.

Decisions to retain or remove items were first made based on the clinical relevance of the item, where items considered less clinically important by a two-thirds majority of authors were removed. Consideration was then given to feasibility (e.g., items that are excessively time-consuming or difficult to view), redundancy between items, and the potential for multiple items to be consolidated into one (e.g., posterior movement of base of tongue and posterior pharyngeal wall contact with base of tongue). Lastly, all items which existed solely for the purposes of skip logic within the Qualtrics version (i.e., items which directed raters to a point further in the VMV based on their response) were removed and that item’s response options consolidated to a related scale (Figure 2—skip logic—original question structure vs. skip logic removed with retained concept.). Skip logic questions contribute to survey structure by allowing only relevant questions to be shown to participants, but their content overlaps with constructs assessed by other items. Removal prevents this overlap from causing issues in statistical analysis. Reducing these items prior to statistical analysis simplified this analysis and allowed analysis to meet statistical assumptions. For example, factor analysis has minimum sample size requirements (100 observations and 5 times the number of cases per items) [25], meaning that factor analysis of 97 items would require a minimum of 485 cases, which was beyond the scope of the current pilot study.

**Figure 3:**
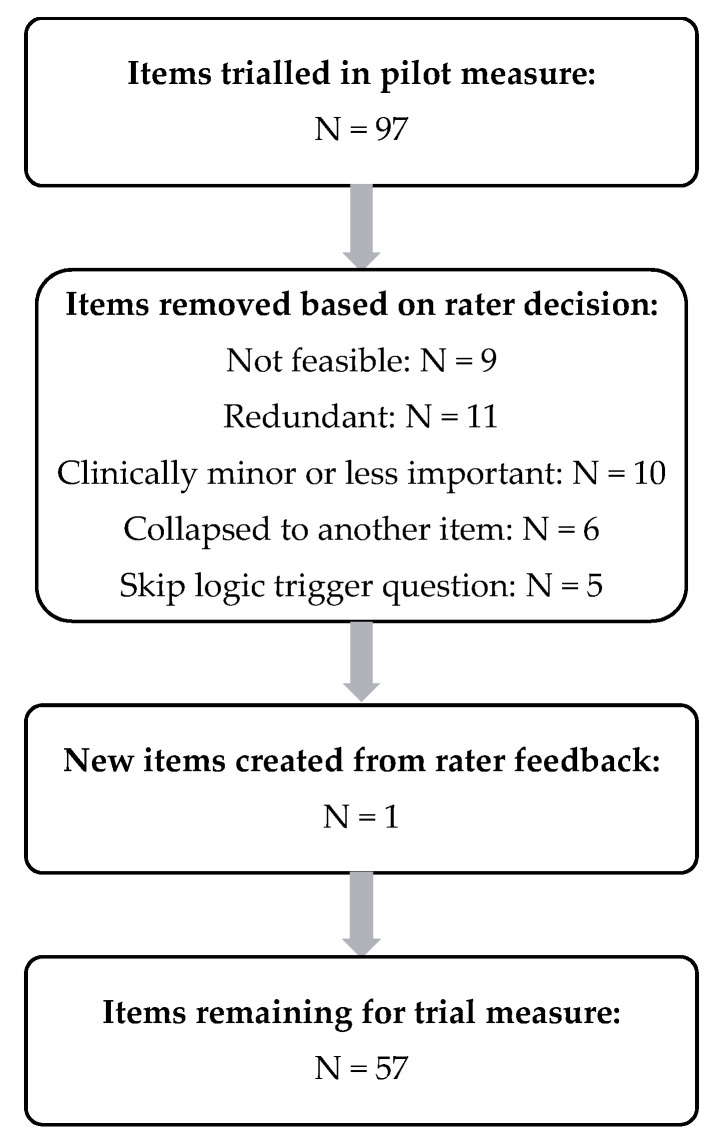
Item reduction from rater feedback.

Item reduction following rater feedback is summarized in Figure 3, Item reduction from rater feedback. Details of items removed and rationales behind these decisions are described in Supplementary Materials, Table S5: Items removed or altered following rater consensus.

Item reduction resulted in the retention of 56 items. One new item, ‘clearing swallow efficacy’, was created with data derived from items rating the volume residue that remained after clearing swallow/s. An overview of items retained per domain is displayed in Supplementary Materials, Table S6: Included items per domain. The researchers then evaluated whether each of the items was clear (i.e., whether it was evident what the item was assessing, whether the manual clearly described what to examine and when to assess) and whether the response scale was adequate (i.e., whether there were too many/too few options in the response scale or ambiguous wording). Of the 57 items evaluated, one was considered unclear and 30 required revisions to their respective response scales.

### 2.7. Psychometric Properties

An analysis of psychometric properties was conducted using the COSMIN taxonomy guidelines [5]. The COSMIN initiative, formulated as a response to the differing terminology found in the literature, developed a unified taxonomy to describe the different measurement properties of instruments [8]. The COSMIN taxonomy was used within this study to define the properties from the domains of reliability and validity, and COSMIN recommendations for the statistical analysis of their quality were also applied [5,11,12,13].

Psychometric properties were determined if the characteristics of the data were appropriate for the intended statistical analysis (i.e., if the assumptions for statistical processing could be met) or if the analyses were feasible for the scope of a pilot study. Psychometric properties included in these analyses were:

- Reliability: The amount of variance in scores which are reflective of true differences in participant function rather than errors in the measure or process of rating [8]. This study included analysis of inter-rater (differences in scores between raters) and intra-rater (differences within a single rater’s scores applied to repeated measures of the same participant).
- Structural Validity: The degree to which the scores adequately reflect the dimensionality of the construct of interest [8]. For example, the VFSS is expected to be multidimensional due to the complexity of the analysis, illustrated by the number of different constructs being assessed (e.g., movement of the bolus, actions of the anatomical structures) and distinct ways constructs are operationalised (i.e., spatial, volume, temporal).
- Internal consistency: The degree of interrelatedness among the VFSS items. Items which measure the same construct should demonstrate a relationship [8]. For example, the items ‘lip seal’, ‘lingual movement’, ‘glossopalatal seal’ and ‘bolus control’ are likely to show a close relationship and score highly on analysis of inter-relatedness as a group.
- Hypotheses testing for construct validity: The extent to which the scores on the measure agree with hypotheses which are theoretically consistent with the condition and the construct being measured [8]. In the case of the VFSS, for example, it is expected that lingual movement correlates strongly with oral residue, with a weaker correlation between lingual movement and parameters of the UES.

Psychometric properties omitted from this analysis were excluded if analysis was not possible, relevant or appropriate for the scope of a pilot. Criterion validity (Diagnostic performance) refers to the degree to which scores adequately reflect a gold-standard measure [8]. This measure for assessment of OD is generally considered to be instrumental assessment [2,26]; however, both FEES and VFSS require the use of visuoperceptual measures to analyse them. There is currently no measure with sufficient evidence of psychometric quality for it to be recommended as a gold-standard for VFSS or FEES [4]. Therefore, criterion validity could not be determined. Cross-cultural validity describes how well a translation of a culturally adapted measure replicates the original [8]. As this is a novel measure, developed only in English and tested in a single geographical location and cultural group, this property was irrelevant. Content validity is the degree to which the content of a measure is an accurate reflection of the construct of interest based on cognate literature and expert opinion [8]. Although not examined in this study, the VMV’s content validity was developed via a Delphi study that is reported in a separate manuscript [16].

Responsiveness refers to a measure’s ability to detect clinically important change over time [8]. Repeated VFSS procedures and assessment pre-post intervention were beyond the scope of the current pilot study. Systematic and random errors in scores that are due to rater or measure errors rather than a true representation of patient change are classed as measurement errors [8]. Statistical analysis requires a total score (summed score) to examine this property, which the pilot measure did not include. Finally, interpretability, the degree to which clinically meaningful connotations can be assigned to the numerical scores or to changes in scores [8], was excluded. Although this is not a psychometric property, its importance is recognised in the COSMIN taxonomy due to the clinical relevance of applying qualitative meaning to quantitative data [5]. This property was not included in this analysis due to the relatively small sample size and the preliminary form of the VMV.

### 2.8. Statistical Analysis

Reliability was analysed using quadratic weighted kappa. The quadratic weighted kappa assesses the degree of disagreement between raters (scale of difference between ordered scorings). Kappa was computed for each rater pair, then averaged to provide a single index of inter-rater reliability [27]. Cronbach’s alpha coefficients were calculated to assess internal consistency for each factor individually as well as for the whole measure. A low Cronbach’s alpha value (alpha < 0.70) indicates inadequate internal consistency, whereas a very high Cronbach’s alpha value (alpha > 0.95) suggests redundancy of items in the factor, which could mean that there are too many items to assess the target construct [28].

With the exception of the inter- and intra-rater reliability analyses, scores from all three raters for 5 mL Thick were used for all analyses as this volume/texture had the largest case numbers available, allowing for statistical assumptions to be met. In the case of inter- and intra-rater reliability, analyses were performed between and within all raters, but were grouped by texture group (i.e., all volumes of Thick were grouped together for analysis). The grouping allowed for comparison of reliability between textures, as swallow behaviours and kinematics may be altered by texture differences [18].

The normality of the dataset will inform the use of parametric or nonparametric statistics. Structural validity was analysed via exploratory factor analysis (EFA) using principal component analysis. Factor analysis is a multivariate technique which identifies the strength of the relationships between items and the underlying latent constructs in the dataset [25]. These latent constructs are referred to as factors, or dimensions. For example, some items in the measure may demonstrate strong relationships with ‘severity’ while others appear related to ‘aetiology’. In EFA, all items are tested for a relationship to every latent construct. A second analysis, known as confirmatory factor analysis (CFA), may be performed to assess whether the model’s factor structure can be replicated. A CFA is only performed if a model of factors is an adequate representation of the theoretical constructs of interest. A CFA was not performed in this study due to the small sample size, which meant that statistical assumptions were not met [25].

Hypothesis testing for construct validity was conducted using Spearman rho correlations and Mann–Whitney U to test the following hypotheses, respectively:

**Hypothesis** **1** **(H1).** *70% of factors will be significantly positively correlated with the FOIS and 5-point ordinal scale*.

**Hypothesis** **2** **(H2).** *No significant differences between genders are expected on any of the item scores*.

## 3. Results

### 3.1. Functional Health Status and Severity Scores

DHI-S scores describe patient severity from self-rating of physical symptoms. Scores ranged from 13–43, with a median of 28.0 (SD ± 7.9, Q1 = 20.0, Q3 = 32.0). Five-point ordinal scale scores ranged from 1–5, with a median of 3.0 (SD ± 1.2, Q1 = 2.0, Q3 = 4.0). FOIS (reversed) scores ranges from 1–7, with a median of 3.0 (SD ± 1.9, Q1 = 2.0, Q3 = 5.0).

### 3.2. Reliability (Intra- and Inter-Rater Reliability)

A quadratic weighted Kappa assessed the degree to which raters produced consistency in the scores they applied between participants, and agreement within scores given to participants on repeated measures [29]. Weighted Kappas between pairs of raters ranged from 0.842 to 0.939, with minor differences between consistencies or views (Table 1. Interrater reliability—Weighted Kappa per Texture). The resulting overall average inter-rater weighted Kappa was in the ‘strong’ range, with an average weighted Kappa of 0.889 [27]. This indicates that raters had a high degree of agreement and suggests that the function or impairment of swallowing as measured by VMV items was coded similarly and consistently across the three raters.

Total intra-rater weighted Kappa on repeated measures of six participants showed excellent intra-rater reliability, resulting in a Kappa of 0.944 (Rater One Mean = 0.948, Rater Two Mean = 0.962, Rater Three Mean = 0.921). Agreement was not calculated between textures due to small data sets. Overall, these results suggest that there is a high level of consistency between raters and that a minimal amount of error was introduced by the independent raters. Ratings were therefore deemed to be suitable to conduct hypothesis testing as outlined before.

### 3.3. Structural Validity

#### Exploratory Factor Analysis

Fifteen items of the 57 retained for the trial measure were excluded from the EFA, as they pertained solely to textures or views (e.g., solids or anterior/posterior) other than 5 mL Thick (lateral view). This resulted in 42 items being assessed in the EFA. The trial using 5 mL Thick was selected for EFA due to this being the trial with the highest number of cases (*n* = 114), being the first trial in the protocol, and thus being best suited to meeting statistical assumptions for EFA. EFA requires a minimum of fives times the number of cases per item [25], and given 114 cases for 5 mL Thick, the maximum number of items permitted for EFA was 22 (22 × 5 = 110). Therefore, the 42 items were divided into two groups.

Item groupings were initially constructed based on theory and clinical reasoning, with items pertaining to anatomically close regions (e.g., oral and oropharyngeal) and/or impairments or events which are closely related (e.g., aspiration and penetration) being grouped. Initial analysis revealed eight factors in both groups. New groupings were created by moving a single item at a time between groups. This process was informed by clinical reasoning, empirical literature and factor loadings (i.e., if a single item was creating a factor by itself, it was moved to another group to attempt to eliminate a one-item factor). The impact of moving single items was evaluated by examining changes in factor loading and total variance, and allowing the items to demonstrate relationships to other items. For example, items related to aspiration or penetration loaded on different factors when items related to the UES functioning were included in the factor analysis, whereas if UES items were excluded from the analyses, both aspiration and penetration items loaded on the same factor.

After the total number of factors was reduced as much as possible, clinical reasoning was used to allocate ambiguous items (i.e., those which loaded approximately equally on more than one factor) to a factor. During this process, three items, ‘Piecemeal Deglutition’, ‘Volume Tracheal residue’ and ‘Coordination of the upper oesophageal sphincter’ were removed due to erratic behaviour (creating weak, theoretically inconsistent single or two-item factors). Finally, the groupings with the combination of items which best represented the most concise and theoretically coherent factors of item loadings were retained. 

This process resulted in two EFA models consisting of five and four factors, respectively (Table 2 and Table 3, Exploratory Factor Analysis). In Group One, five factors explained 71.8% of the total variance, with most items loading on Factors One and Two. Factor One explained 18.6% of the variance, indicating multidimensionality, as Factor One accounted for <20% of the variability and the ratio of the variance from Factor One to Two is less than four [30]. The Group Two factor loadings reflected similar findings. A four-factor solution explained 77.4% of the total variance, with most items loading on Factors 6 and 7 (factors are named with sequential numbers continuing from group One to Two), and a ratio of less than four in variance between the two factors. These findings also suggest multidimensionality.

### 3.4. Internal Consistency

Cronbach’s alpha was calculated per factor and for the whole measure for 5 mL Thick data on the 39 items retained from the EFA results, following the removal of three items creating erratic behaviour (Table 4. Internal Consistency). Scores for all factors and overall were adequate (Cronbach’s alpha >0.70 and <0.95), except for Factor Four (0.698) [28].

### 3.5. Hypothesis Testing for Construct Validity 

The data were not normally distributed, therefore nonparametric correlations were calculated.

*Hypothesis One*, which stated that factor scores will be significantly positively correlated with FOIS and 5-point ordinal scale scores in 70% of factors, was partially supported (Table 5. Factors’ correlation with FOIS and 5-point ordinal scale). The hypothesis was partially supported with a weak to moderate positive correlation (FOIS mean: 0.171, range = −0.157–0.415; 5-point ordinal scale mean: 0.199, range −0.055–0.432) that was statistically significant in seven of nine (77%) factors, and positive but non-significant in one (Factor 8: Penetration) [31,32]. The factor containing UES Function items generated weak inverse correlations (−0.157 and −0.055).

Hypothesis 2, which stated that there will be no significant difference on item scores between genders, was supported. The hypothesis was supported by a Mann–Whitney U test, which found no significant difference between the scores of male and female patients: Mean Rank_Male_ = 2393.28 (Sum of Ranks = 7,237,279.00); Mean Rank_Female_ = 2396.59 (Sum of Ranks = 4,227,587.00); *U* = 2,663,479.00; *p* = 0.932, two-tailed.

## 4. Discussion

The psychometric properties of the pilot VMV were evaluated in this study. The analysis was conducted with reference to a classical test theory (CTT) psychometric paradigm and the COSMIN framework [9,10,11,12,13]. CTT is well-suited for initial investigations of psychometric properties [33] and is useful in measure development, as many constructs of interest are not directly observable in health practice. For example, laryngeal vestibule closure may be purported to be assessed by VFSS analysis; however, ‘closure’ is not directly measured. The clinician’s perception of the proximity of pixels produced by digitisation of fluoroscopy is the observable data. Clinicians assign meaning to this ‘proxy indicator’ to measure the unobserved construct of ‘closure’. CTT-informed analysis determines the success of the proxy indicator in measuring the unobservable phenomenon [34]. A key tenet of CTT is that the scores of each item are produced by a combination of the unobservable ‘true’ score, summed with the unavoidable errors and biases introduced by the use of a proxy indicator. Errors in CTT are assumed to be random and unique to each item [34]. The COSMIN framework was used to define the psychometric terms applied and to guide the statistical methodology used [11,12,13].

Statistical analysis found that the inter-rater reliability coefficients of the VMV were in the ‘strong’ range overall and included scores in the excellent range between Raters One and Two. This indicated that the target concepts were clearly and consistently understood between the three raters from different professions—speech-language pathology (SLP), radiology and phoniatrics. This was reflected in the item reduction process, where the majority of items selected for the next version of the measure were considered ‘clear/unambiguous’ by all raters. Intra-rater reliability was excellent, indicating that the pilot VMV supports a consistent internal schema within raters that is stable across time [27].

Structural validity analysis via EFA produced a 5-factor and 4-factor solution. Group One contained variables primarily relating to swallowing events and kinematics occurring superiorly in the oropharyngeal tract and early in the swallowing process (e.g., hyoid movement). Group Two resulted in items pertaining to laryngeal, hypopharyngeal and late-stage events (e.g., residue post swallow). However, some items behaved erratically (i.e., ‘piecemeal deglutition’ caused a factor with a single item loading on it) and some items had ambiguous loadings (e.g., ‘Oropharynx residue volume’ loaded similarly on two factors). This is likely related to sample size. Items with ambiguous loadings were allocated to a group and factor based on theoretical consistency of the grouping (e.g., oropharynx residue volume was grouped with the factor containing ‘oral residue volume’ as opposed to the factor containing ‘location of material at swallow initiation’, as the pairing with another item measuring residue, rather than a temporal event, is more logically consistent). Three items, ‘Piecemeal deglutition’, ‘Volume tracheal residue’ and ‘Coordination of the upper oesophageal sphincter’ were removed as they created single item factors or groupings which were illogical. Therefore, the groups represent preliminary proposals at this time; conclusive evidence of factor structure will require greater numbers of participants.

EFA indicated that the measure is multidimensional, meaning that the construct under assessment has two or more dimensions. In VFSS, a simple construct such as velum movement may be unidimensional (i.e., the underlying dimension of the construct is velum elevation). A multidimensional construct might be aspiration, where the dimensions contributing to the construct include volume of aspirate, time when aspiration occurs, and the patient’s awareness of the event. In the context of the pilot VMV, this finding means that visuoperceptual examination of VFSS likely involves multiple underlying dimensions. However, this needs to be confirmed in a larger sample with an EFA that includes all items in a single analysis (as opposed to split into two groups) followed by a confirmatory factor analysis. Total percentage of variance explained was >70% for both models, indicating that random error was not excessive [35].

Internal consistency was good (alpha > 0.7 but < 0.95) for 8 of the 9 factors and overall, with only one factor (Factor Four, which contained items pertaining to premature spillage and swallow initiation) not reaching this zone alpha by only 0.002 [28]. This indicates good content coverage, but item reduction may be possible to streamline the measure. Further analysis of the preliminary measure using the Rasch measurement model (RMM), a type of item response theory, would provide additional information about the dimensionality, differential item functioning, person-ability scores, and item difficulty scores. This would assist in identifying items that do not meet RMM person and item fit criteria and could subsequently be discarded [33].

Hypothesis testing for convergent validity tested two hypotheses. The first, an expected positive relationship between VMV and both FOIS and 5-point ordinal scale scores was partially supported. All but one factor had a weak, positive statistically significant correlation. That is, as the degree of impairment increased (as measured by texture prescription) and the radiologist’s perception of overall severity of OD increased, so did scores on the VMV. The factor containing the UES items was negatively correlated with both FOIS and the 5-point ordinal scale. It might be expected that the UES, as the terminal part of the pharynx, would reflect dysfunction from superior abnormalities of the oral cavity, pharyngeal shortening and constriction, cervical spine and hyolaryngeal function [36]. However, the inverse correlation indicates that this was not the case in this pilot. This finding may be a related to the small sample size or the texture/volume analysed; 5 mL Thick may not be ideal to reveal UES deficits because the small volume is less likely to be problematic for passage through the UES, given that larger thick volumes produce greater durations of opening, amplitudes of relaxation and earlier opening onset (i.e., thick volumes induce greater challenges to the swallow system) [37]. The inverse correlation result may also be related to the construct itself. For instance, the UES items were the only items where ‘opening’ was measured, while other items assess contact with other structures, volumes of material and timing of kinematics. As this was a pilot study, explanation of this finding cannot be conclusive. Further analysis in a larger sample is required.

The second hypothesis, a lack of association between scores on VMV and gender, was supported. This result was expected given that OD severity as perceptually analysed on VFSS should have no association with gender [38]. These two findings indicate that it is likely that the VMV is measuring the target construct. Finally, a review by the authors of the feasibility, clinical relevance and redundancy of the items found that approximately half of the items could be removed. This is expected in measure construction, where multiple items may assess the same construct in the pilot and then the most suitable are retained following initial testing. Removal of items also assists in developing a measure’s suitability for clinical use; the pilot iteration of the measure was excessively time consuming, taking over 40 min for analysis. A measure useful for practice must balance adequate content coverage with feasible administration time.

The pilot VMV exhibits evidence of content validity [16], intra- and inter-reliability, structural validity, internal consistency and hypothesis testing. In a psychometric review of current visuoperceptual VFSS measures, only nine measures were found where evidence of the scale’s validity and reliability were reported. The quality of the reported psychometric properties was limited, primarily due to unclear reporting and methodological flaws. [4]. The VMV represents the first visuoperceptual measure for VFSS that has been constructed with reference to the international best practice guidelines of the COSMIN initiative [10,39]. The VMV has evidence of its robust content validity, established through an extensive international Delphi process involving 50 experts from 27 countries [16]. In addition, this measure was piloted using raters from three different disciplines (SLP, radiology and phoniatrics) and their expertise informed measure refinement and item reduction. No other measure has utilised such comprehensive and robust methodology [4]. Similarly, initial evidence of the VMV’s structural validity and dimensionality was provided through the EFA results.

### Limitations and Future Research

Limitations of this study are the small sample size, which resulted in the reliability and EFA analyses being limited to 5 mL Thick to meet statistical assumptions. The study was conducted at a single site, and while the population was reasonably heterogenous, the sample does not comprehensively reflect all possible aetiologies and comorbidities of the OD population. The analysis was conducted using only a CTT framework, which is known to have a number of limitations. For example, each item’s score is comprised of its ‘true’ score and random error in CTT, and as the distribution of the error is random around a mean of zero, errors from different items will generally negate each other. This means that scales which include many items may yield disproportionately strong reliability [34]. However, the application of CTT represents a first step in the psychometric evaluation of the VMV. The combination of CTT with another theoretical framework, such as the RMM, would yield further valuable insights about the measurement properties of the VMV [12,40]. In addition, some psychometric properties (e.g., test re-test, measurement error) and interpretability were out of the scope of this study. Finally, this study reports on a pilot version of the VMV that is not yet ready for formal clinical use. It is anticipated that future studies involving larger patient populations will allow additional statistical analysis (e.g., EFA and RMM analysis including all items), investigation of additional psychometric properties, and investigation using psychometric paradigms that complement each other (i.e., CTT and IRT). Together, these will help create a refined version of the preliminary VMV which is suitable for clinical use.

## 5. Conclusions

The CTT analysis indicates that the initial psychometric properties of a pilot version of the VMV may be adequate for analysing VFSS in a valid and reliable manner. The VMV appears to have good inter and intra rater reliability. The VMV is multidimensional, based on EFA results, and exhibits good internal consistency. Hypothesis testing for construct validity indicates that the relationship between OD severity and population characteristics is as expected, with VMV severity scores increasing as functional severity on other measures increase. Future studies of the preliminary VMV with larger samples and additional statistical analysis using the RMM is recommended as this will add to the psychometric evidence of the VMV. The VMV pilot study represents the first step in developing a robustly validated measure for visuoperceptual analysis of VFSS which is intended to be suitable for research and clinical purposes in its final version.

## Figures and Tables

**Table 1:** Interrater reliability—Weighted Kappa per Texture.

	Rater One vs. Two	Rater One vs. Three	Rater Two vs. Three	Average
Thick (L3)	0.932	0.886	0.882	0.900
Thin (L0)	0.930	0.866	0.860	0.885
Pudding (L4)	0.939	0.874	0.868	0.894
Solids (L7)	0.934	0.892	0.852	0.893
Anterior-Posterior view	0.868	0.910	0.842	0.873
Total (Average)	0.921	0.886	0.861	0.889

**Table 2:** Exploratory factor analysis—factor loadings of model one.

Item No.	Item Descriptor	Factor 1	Factor 2	Factor 3	Factor 4	Factor 5	Communalities
1.1	Number of swallows	0.120	**0.867**	0.126	−0.060	−0.105	0.797
1.2	Lingual motion (liquids)	**0.748**	0.091	0.349	0.088	−0.053	0.701
1.3	Bolus formation (liquids)	**0.777**	0.019	0.112	−0.156	0.132	0.659
1.4	Bolus transport (liquids)	**0.728**	0.194	0.069	0.138	−0.236	0.647
1.5	Base of tongue retraction	**0.620**	0.086	0.090	0.412	−0.071	0.574
1.6	Velum elevation	**0.530**	−0.052	0.413	0.120	−0.099	0.478
1.7	Premature Spillage location	0.239	0.056	0.169	**0.781**	0.145	0.719
1.8	Location material at swallow initiation	−0.037	−0.067	0.057	**0.820**	0.002	0.681
1.9	Hyoid excursio—superior movement	0.245	0.079	**0.827**	0.081	−0.162	0.783
1.10	Hyoid excursion—anterior movement	0.233	0.286	**0.793**	0.023	0.094	0.774
1.11	Laryngeal excursion	0.104	0.071	**0.898**	0.170	−0.136	0.870
1.12	Pharyngeal constriction pharyngeal obliterated space	0.225	**0.571**	0.297	0.279	0.001	0.543
1.13	Clearing Swallow—Location of residue when swallow triggered	−0.047	**0.904**	0.088	−0.021	0.102	0.837
1.14	Clearing swallows (number)	−0.155	**0.927**	0.115	−0.109	0.097	0.917
1.15	Width of UES opening	−0.032	0.124	−0.072	−0.015	**0.922**	0.872
1.16	UES closure impedes flow	−0.185	0.037	−0.113	0.110	**0.909**	0.886
1.17	Oral residue volume	**0.629**	−0.063	0.262	0.350	−0.276	0.666
1.18	Oropharynx residue volume	**0.559**	0.008	0.054	0.593	−0.050	0.670
1.19	Valleculae residue volume	0.383	**0.592**	−0.148	0.078	0.196	0.564
Eigenvalue		3.525	3.282	2.704	2.139	1.989	
% of Total Variance		18.55	17.27	14.23	11.26	10.47	
Total variance		71.78%					

The bold represent the proposed models for the loading on these factors.

**Table 3:** Exploratory factor analysis—factor loadings model two.

Item No.	Item Descriptor	Factor 6	Factor 7	Factor 8	Factor 9	Communalities
2.1	Laryngeal vestibule closure (LVC)—base to arytenoids	0.248	**0.754**	0.160	0.191	0.691
2.2	Epiglottic tilting	0.148	**0.716**	0.036	0.259	0.604
2.3	Laryngeal vestibule closure—base to arytenoids contact relative to UES opening	0.136	**0.722**	−0.102	0.212	0.595
2.4	Pharyngeal wall movement	−0.051	0.361	−0.307	**0.400**	0.387
2.5	Aspiration present on x number of swallows	0.233	**0.835**	0.012	−0.003	0.751
2.6	Response to aspiration	**0.978**	0.171	0.086	0.052	0.995
2.7	Cough ability to eject material	**0.952**	0.166	0.066	0.097	0.948
2.8	Cough latency (ordinal)	**0.962**	0.167	0.070	0.088	0.966
2.9	Aspiration occurrence timing	**0.955**	0.168	0.098	0.013	0.950
2.10	Aspiration volume	**0.978**	0.171	0.086	0.052	0.995
2.11	Penetration present on x number of swallows	0.160	−0.065	**0.839**	0.179	0.765
2.12	Penetration occurrence timing	0.147	0.026	**0.907**	0.053	0.848
2.13	Penetration depth	0.018	0.478	**0.757**	0.217	0.849
2.14	Response to penetration	0.167	**0.772**	0.485	0.143	0.881
2.15	Permanence of penetration	0.132	**0.723**	0.523	0.164	0.841
2.16	Post. pharyngeal wall of hypopharynx residue volume	0.138	0.161	0.089	**0.674**	0.507
2.17	Pyriform sinus residue volume	0.052	0.088	0.153	**0.872**	0.794
2.18	Laryngeal surface epiglottis residue volume	**0.582**	0.369	0.350	0.032	0.598
2.19	Laryngeal vestibule residue volume	0.164	**0.799**	−0.010	0.043	0.667
2.20	Clearing Swallow Efficacy	0.018	0.212	0.214	**0.869**	0.845
Eigenvalue		5.300	4.777	2.971	2.432	
% of Total Variance		26.48	23.89	14.86	12.16	
Total variance		77.38%				

The bold represent the proposed models for the loading on these factors.

**Table 4:** Internal Consistency.

Factors and Description of Items within the Factor	Cronbach’s Alpha
Factor 1. Lingual control and motion, velum motion, oral and oropharynx residue	0.810
Factor 2. Number of swallows, clearing swallows and pharyngeal contraction	0.861
Factor 3. Hyoid and larynx movement	0.876
Factor 4. Premature spillage and swallow initiation	0.698
Factor 5. Upper oesophageal sphincter function	0.836
Factor 6. Aspiration and underside epiglottis residue	0.934
Factor 7. Epiglottis movement, aspiration, penetration permanence and response and laryngeal vestibule closure	0.873
Factor 8. Penetration	0.853
Factor 9. Pharyngeal wall movement, pharyngeal residue and clearing swallows	0.714
Total Measure	0.902

**Table 5:** Factors’ correlation with FOIS and 5-point ordinal scale.

Factor		FOIS	5-Point Scale
		Correlation coefficient	Correlation coefficient
1	Lingual control and motion, velum motion, oral and oropharynx residue	0.228 **	0.226 **
2	Number of swallows, clearing swallows and pharyngeal contraction	0.289 **	0.324 **
3	Hyoid and larynx movement	0.415 **	0.432 **
4	Premature spillage and swallow initiation	0.185 **	0.264 **
5	Upper oesophageal sphincter function	−0.157 *	–0.055
6	Aspiration and underside epiglottis residue	0.199 **	0.225 **
7	Epiglottis movement, aspiration, penetration permanence and response and Laryngeal Vestibule closure	0.231 **	0.234 **
8	penetration	0.063	0.058
9	Pharyngeal wall movement, pharyngeal residue and clearing swallows	0.086 **	0.086 **

* Correlation is significant at the 0.05 level (2-tailed). ** Correlation is significant at the 0.01 level (2-tailed).

## Data Availability

The data presented in this study are available on request from the first author. The data are not publicly available due to conditions of approval from the governing Human Research Ethics Committee.

## Supplementary Materials

The following supporting information can be downloaded at: https://www.mdpi.com/article/10.3390/jcm11030724/s1, Table S1: Aetiology of Oropharyngeal Dysphagia; Figure S1: VFSS protocol; Table S2: Administration protocol; Table S3: Functional Health Status and Severity measures; Table S4: Functional Health Status and Severity measures scoring; Table S5: Items removed or altered following rater consensus; Table S6: Included items per domain.
