# Supplementary material for: A Visuoperceptual Measure for Videofluoroscopic Swallow Studies (VMV): A Pilot Study of Validity and Reliability in Adults with Dysphagia
Source: J Clin Med. 2022 Jan 29;11(3):724. doi: 10.3390/jcm11030724 (PMC8837107; doi:10.3390/jcm11030724)
Supplement: Supplementary file 1 [file jcm-11-00724-s001.zip › jcm-1544116-supplementary.pdf]

Journal of
Clinical Medicine

MDPI

Supplementary Materials

Table S1. Aetiology of Oropharyngeal Dysphagia.

Category	Age & Gender	Details		N	Percent
Cancer	Med: 70, Range: 56 – 91, Q1 = 62.0, Q3 = 77.5)<br>M = 12, F = 5	• Oral<br>• Pharyngeal<br>• Laryngeal<br>• Tonsillar<br>• Skull base	• Floor of mouth<br>• Tongue base<br>• Oesophageal	17	43.6
Neurological disorder	Med: 39.5, Range: 21 – 78, Q1 = 27.25, Q3 = 68.25)<br>M = 5, F = 5	• Motor Neuron Disease<br>• Idiopathic bulbar symptoms / movement disorder	• Incomplete paraplegia<br>• Guillain Barré Syndrome<br>• Myasthenia Gravis<br>• Muscle hypotonia	10	25.6
Surgery	Med: 65, Q1, Range: 49 – 79, = 62.0, Q3 = 74.0)<br>M = 5, F = 2	• Thyroplasty<br>• Laser base of tongue resection	• Thyroidectomy<br>• Tracheal resection<br>• Pneumonectomy	7	17.9
Anatomical abnormality	Med: 78, Range: 57 – 89, Q1 = 57.5, Q3 = 84.0)<br>M = 3, F = 2	• Zenker's diverticulum<br>• Cervical spine trauma	• Stenosis of Upper Oesophageal Sphincter	5	12.8

Med: Median, Q1: Quartile 1, Q3: Qurtile 3, M: Male, F: Female

Figure S1. VFSS protocol.

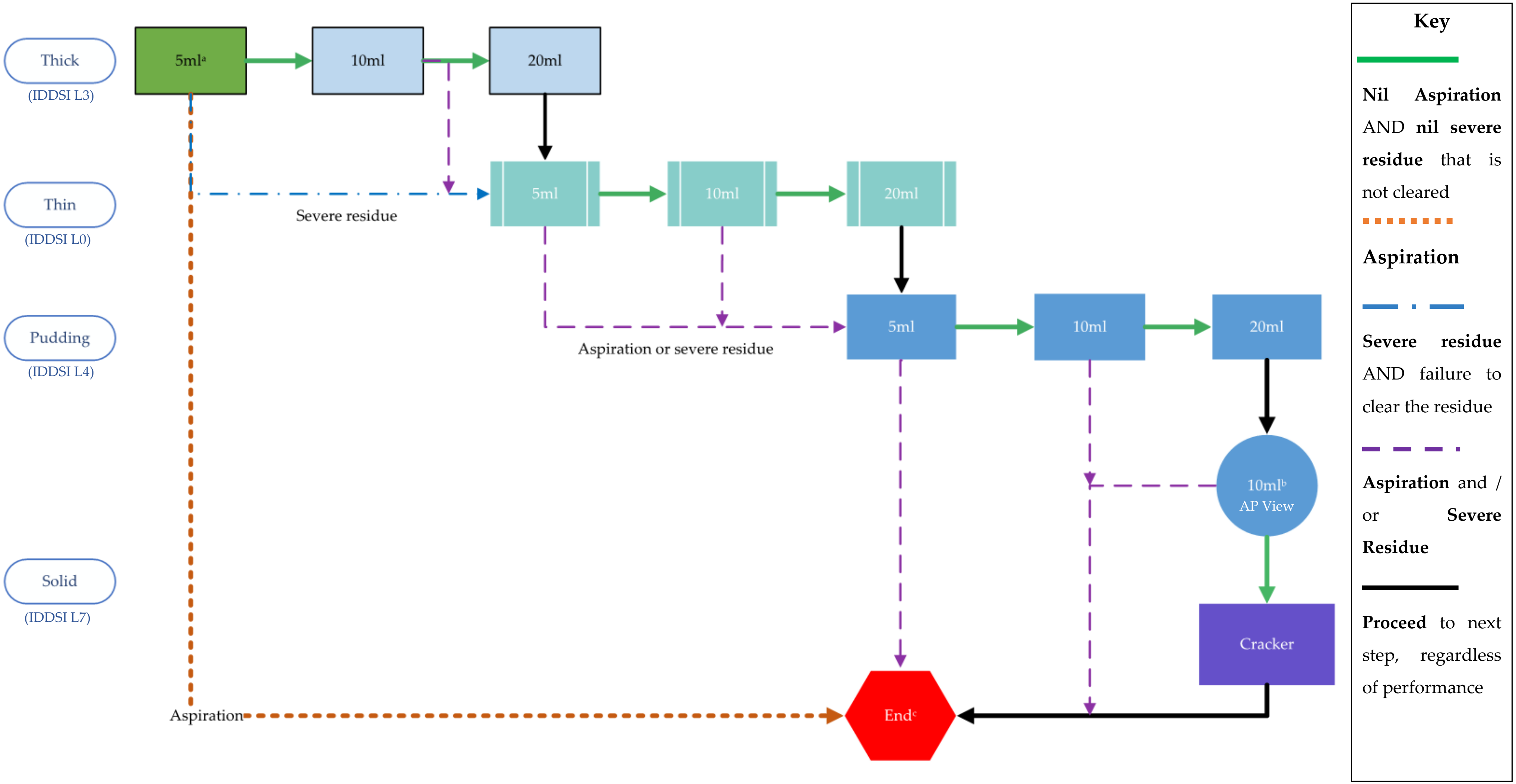

[a] Start, [b] Anterior posterior view [c] Additional trials as per clinician judgement / local processes if required (e.g. testing specific compensatory postures) commences

Table S2. Administration protocol.

Equipment

- 2 x 10 ml slip tip syringes
- 3 x 20 ml slip tip or catheter tip syringe

Contrast preparation

Mix water with contrast (Omnipaque or Accupaque) and thickener (Nutilis Clear) according to the following recipe ratios. Wait at least 10 mins to thicken in room temperature (~20°C).

	Volume of Water (ml)	Mass of thickener (g)	Volume of contrast (ml)	Comments on preparation	Pressure mPa·s	IDDSI level
**Thick**	25	0.85	25	1. Water + Contrast 2. Thickener 3. Stir till desired thickness	250	3
**Thin**	25	0	25	-	2.3	0
**Pudding**	25	2.31	25	1. Water + Contrast 2. Thickener 3. Stir till desired thickness	800	4
**Cracker:** x 1						7

Delhaize mini toast[1] – cover one face with 2ml thick spread pudding contrast.

Patient position

Lateral: Seated, head strict lateral. Shoulder turned approximately 5-10 degrees to posterior oblique.

Anterior-Posterior: Seated, head midline.

Lateral view

The visualization field includes anteriorly the lips, superiorly the nasal cavity, posteriorly the cervical spine, the complete larynx and inferiorly the pharyngoesophageal segment as well as the upper cervical oesophagus.

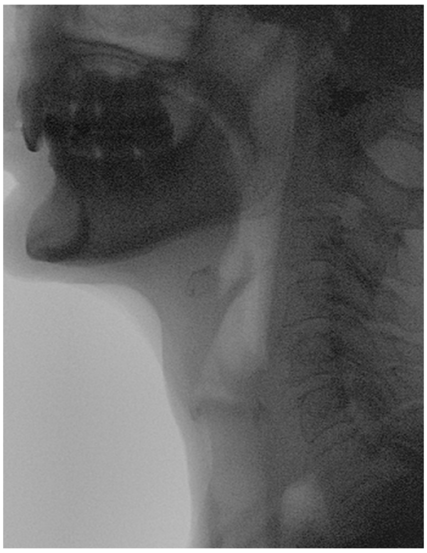

Recommended Lateral Viewing Field
Image credit: St John of God Health Care

Exposure setting

Continuous or 30 or 25 pulses per second.

Image capture

Maximum temporal resolution - 30 fps or 25fps [21].

Contrast delivery

Liquids/Pudding:

- 5 and 10 ml: utilise 10ml syringe; draw up prepared contrast and then place requisite amount on anterior tongue.
- 20 ml: utilise 10ml syringe; draw up prepared contrast and then place requisite amount on anterior tongue.

Instructions:

- Instruct patient to open mouth:
  "Please open your mouth. I will put some liquid on your tongue. Hold it in your mouth until I say swallow."
- Place contrast syringe under centre of tongue. Instruct patient to close mouth around syringe. Taking approx. 3 seconds, gently depress plunger.
- Commence exposure when clinician has moved out of field.
- Instruct patient to swallowing by saying: 'Swallow now'.

Cracker:

- Ensure cracker covered well in pudding contrast.

Instructions:

- Instruct patient to place whole cracker in mouth: "Put the whole cracker in your mouth. Do not bite it. Do not start chewing until I tell you to."

[1] Not for participants with Gluten, Wheat, Eggs, Milk, Sesame seeds allergies.

Table S3. Functional Health Status and Severity measures.

Measure	Description	Scoring/Administration
Deglutition Handicap Index, Symptom subscale (DHI-S) [23]	10 items describing symptoms of OD (e.g., coughing, choking, drooling)	Participant self-report.<br>Each item scored on a 5-point scale (0 – 4; resp. never – always) to represent frequency of symptom.<br>Range of sum score: 0 – 40.
5-point ordinal severity scale	Single item with 5-point response scale to indicate clinician's overall impression of OD severity based on viewing VFSS.	Completed by radiologist after VFSS (ranging from 'normal swallowing' to 'not able to swallow').
Functional Oral Intake Scale (FOIS) [24]	Single item with 7-point response scale to describe patient's food/fluids texture restrictions, feeding methods or nutritive intake.	Completed by clinician after VFSS (Response scale ranged from 'nothing by mouth' to 'total oral diet with no restrictions').<br>FOIS scores were reversed so that higher scores indicated a higher degree of impairment, for consistency with the other outcome measures and the VMV.

Table S4. Functional Health Status and Severity measures scoring.

DHI – Symptom Subscale	Scoring
	**5-point scale per item**
I feel discomfort when I swallow	**1** = never
Food or liquids gets stuck or blocks my throat	**2** = almost never
I have difficulty swallowing liquids	**3** = sometimes
I cough or clear my throat during or after a meal	**4** = almost always
I choke when eating or drinking	**5** = always
I feel like food or liquids come back up after a meal	
I have difficulty chewing	
Food or liquids go through my nose when I drink or eat	
I drool	
My throat hurts when I swallow	
**5-point ordinal scale**	**Scoring**
	**Single score selected**
Normal	**1**
Minor problems	**2**
Moderate problems	**3**
Severe problems	**4**
Not able to swallow	
**FOIS reversed**	**Scoring**
	**Single score selected**
Total oral diet with not restrictions.	**1**
oral diet with multiple consistencies, but requiring special preparation or compensations.	**2**
Total oral diet with multiple consistencies, but requiring special preparation or compensations.	**3**
Total oral diet of a single consistency.	**4**
Tube dependent with consistent oral intake of food or liquid.	**5**
Tube dependent with minimal attempts of food or liquid.	**6**
Nothing by mouth	**7**

Table S5. Items removed or altered following rater consensus.

Items	Operationalisation	Reason for removal / modification	Comments from raters	Action
• Location of swallow initiation<br>• Laryngeal vestibule closure (epiglottic body / tip contacts arytenoids)<br>• Laryngeal vestibule closure (base of epiglottis to arytenoids)<br>• Duration to laryngeal vestibule closure (hyoid burst to tip/body epiglottis contact to arytenoids)<br>• Duration to laryngeal vestibule closure: (hyoid burst to tip/body epiglottis contact to arytenoids)<br>• Cough - latency until cough / throat-clear<br>• UES coordination - time from glossopalatal junction opening to UES opening<br>• UES opening duration	Frame counting	Not feasible	• Time consuming compared to visuoperceptual ordinal scales<br>• No compelling evidence of validity	Deleted
• Tracheal residue (depth below vocal folds)	Ordinal scale		• Operationalisation problematic<br>• Concept captured in residue volume	
• Base of tongue to posterior pharyngeal wall approximation<br>• Glossopalatal seal (liquids, during bolus hold)	Ordinal scale	Redundant with another item	• Redundant with base of tongue movement<br>• Redundant with premature spillage	Deleted

Items	Operationalisation	Reason for removal / modification	Comments from raters	Action
• Piecemeal deglutition (number of subdivisions)			• No evidence that counting is clinically meaningful at this time (e.g., is seven meaningfully worse than five). Presence or absence important. Concept also captured in number of swallows.	
• Location of swallow initiation (latency past ramus of mandible)			• Redundant with 'Location of swallow initiation' (region of head of bolus at swallow initiation)	
• Laryngeal vestibule closure (duration)			• Redundant with 'Bolus entry to UES in relation to Laryngeal vestibule closure'	
• Laryngeal vestibule closure (duration of arytenoid to base of epiglottis contact)			• Duplicated concept	
• Pharyngeal constriction (contact of base of tongue and velum with the walls of the pharynx)			• Redundant with base of tongue / posterior pharyngeal wall	
• Penetration (location of source of penetration)			• Concept is captured in timing of aspiration (i.e. penetration before swallow means superior source)	
• Cough / throat clear (consistency of clearing ability)			• Overlap with 'Success in ejecting material'. Likely less suitable for VFSS; patient unable to clear once is significant, repeat ratings not required	

Items	Operationalisation	Reason for removal / modification	Comments from raters	Action
• Aspiration (location of source of aspiration)			• Captured in timing of aspiration and residue post swallow	
• Nasopharynx misdirection (time)<br>• 3 x items re: of premature spillage (volume)<br>• Latency of glossopalatal junction opening to laryngeal vestibule closure (from when epiglottic body / tip contacts arytenoids)<br>• Latency glossopalatal junction opening to Laryngeal vestibule closure (from when epiglottic base contacts arytenoids)<br>• Anterior - posterior pharyngeal contraction (asymmetrical wall movement<br>• Anterior - posterior pharyngeal contraction (symmetrical wall movement)<br>• Oral cavity residue (location)<br>• Aggregation of solids (volume)	Ordinal scales	Clinically less important	• Relatively rare<br>• Volume not as important as present / absent<br>• UES to laryngeal vestibule closure more important construct to capture<br>• UES to laryngeal vestibule closure more important construct to capture<br>• Rarely used<br>• Rarely used<br>• Removed locations material present oral cavity – now captured in present / absent item<br>• Insufficient evidence that volume is clinically meaningful	Deleted

Items	Operationalisation	Reason for removal / modification	Comments from raters	Action
• Time when arytenoid cartilage to epiglottic base contact occurs in relation to the bolus entry to the UES	Ordinal scales	Collapsed to another item	• Could be merged with another laryngeal vestibule closure scale	Concept retained in others measures of laryngeal vestibule closure through scale change
• Clearing swallow volume of residue in five different anatomical zones<br>• Residue locations	Heat map		• Could be captured with small ordinal scales	Heat map options collapsed to ordinal scales

Table S6. Included items per domain.

Domain	Item names[a]	Example Item and Rating Scale
Oral Bolus Transit - Liquids	• Lingual motion (liquids)<br>• Bolus formation (liquids)<br>• Bolus transport (liquids)<br>• Base of tongue retraction<br>• Velum elevation<br>• Oral residue volume<br>• Oropharynx residue volume	**Lingual motion (liquids):**<br>*Select option which best describes the FIRST swallow.*<br>o 5: The tongue tip rises to contact the alveolar ridge. Tongue elevation progresses through the body of the tongue from anterior to posterior, squeezing the bolus along the hard palate.<br>o 4: Lingual action as per '5', but there is 'hesitation' prior to the initiation of movement.<br>o 3: Lingual action as per '5', with repetitive lingual hesitations or discoordination.<br>o 2: Lingual movement is present, but appears abnormal in some respects (discoordination or hesitation) and the tongue does not contact the hard palate.<br>o 1: Nil or minimal lingual movement is present and the tongue does not contact the hard palate
Oral Bolus Transit - Solids	• Lingual movement with solids<br>• Posterior movement of solids<br>• Timeliness of solid bolus transfer<br>• Aggregation of solids	**Posterior movement of the solid bolus:**<br>o 5: The whole bolus moves towards the pharynx.<br>o 4: More than half or the bolus moves towards the pharynx.<br>o 3: Slightly less than half of the bolus moves towards the pharynx.<br>o 2: Minimal fragments of the bolus move towards the pharynx.<br>o 1: None of the bolus moves towards the pharynx.

Nasopharyngeal seal	• Nasopharynx misdirection	**Volume of material which enters the nasopharynx:**<br>*If multiple swallows, select option which describes worst performance.*<br>o 4: Minimal: a line of contrast coats the posterior pharyngeal wall, at the level of the hard palate or above.<br>o 3: Mild: a line of contrast coats the superior surface of the velum. The posterior pharyngeal wall, at the level of the hard palate or above, may also be coated.<br>o 2: Moderate: Material coats the superior surface of the velum, the posterior pharyngeal wall and / or the superior surface of the hard palate (interior of nasopharynx).<br>o 1: Substantial: A large portion of the bolus moves into the nasopharyngeal cavity resulting in a substantial volume present on the superior surface of the hard palate (interior of nasopharynx).
Pharyngeal Bolus Transit	• Number of swallows<br>• Piecemeal Deglutition<br>• Number of clearing swallows<br>• Clearing Swallow - Location of residue when swallow triggered<br>• Pharyngeal constriction pharyngeal obliterated space<br>• Valleculae residue volume	**Piecemeal deglutition:**<br>o 2: No subdivision of contrast material in the oral cavity; contrast material is swallowed as one whole bolus.<br>o 1: Contrast material is subdivided in the oral cavity and swallowed in two or more subsequent swallows.

Hyo-laryngeal Movement	• Hyoid excursion - superior movement<br>• Hyoid excursion - anterior movement<br>• Laryngeal excursion	**Hyoid excursion - superior movement:**<br>*Select option which best describes hyoid movement of FIRST swallow. Ensure rating is from the lowest possible position of the hyoid (i.e. the relaxed, most inferior possible position)*<br>o 4: Substantial difference in position between resting position and maximal superior position; hyoid appears to move more than the equivalent height of one cervical vertebra.<br>o 3: Moderate difference in position between resting position and maximal superior position; appears to move the equivalent height of one cervical vertebra.<br>o 2: Slight difference in position is visible between resting position and maximal superior position; appears to move less than the equivalent height of one cervical vertebra.<br>o 1: Minimal or no difference in position visible between resting position and maximal superior position.
Premature Spillage and swallow Initiation	• Premature spillage location<br>• Location material at swallow initiation	**Premature spillage location: Presence of contrast material in pharynx prior to velum elevation on bolus hold task – select the most inferior region material is present in during frame immediately prior to velum elevation:**<br>*Select option which best describes the FIRST swallow.*<br>o 7: Nil contrast material is present posterior of the velum.<br>o 6: Upper oropharynx, posterior of velum.<br>o 5: Valleculae.<br>o 4: Upper hypopharynx.<br>o 3: Pyriform sinus.<br>o 2: Laryngeal vestibule<br>o 1: Trachea

Upper Oesophageal Sphincter	• Width of Upper Oesophageal Sphincter opening<br>• Upper Oesophageal Sphincter closure<br>• Coordination of The Upper Oesophageal Sphincter	**Width of Upper Oesophageal Sphincter opening**<br>**Rate the frame where the UES is open at its widest.*<br>○ 3: UES opening is wide, and permits most of the bolus in the hypopharynx to pass without impedance.<br>○ 2: Width of UES opening is reduced – it may be unevenly narrow in one or more segments of the UES – but permits a substantial portion of bolus to pass without impedance.<br>○ 1: Width of UES opening is impeded or restricted – minimal bolus passage past the constriction is possible.
Aspiration	• Response to aspiration<br>• Cough ability to eject material<br>• Cough latency (ordinal)<br>• Aspiration timing<br>• Aspiration volume<br>• Laryngeal surface epiglottis residue volume	**Aspiration timing** **select all that apply*<br>○ During swallow<br>○ Before swallow initiated<br>○ After swallow completed

Laryngeal Vestibule Closure Function	• Laryngeal vestibule closure (LVC)- base to arytenoids<br>• Epiglottic tilting<br>• Laryngeal vestibule closure - base to arytenoids contact relative to Upper Oesophageal Sphincter opening<br>• Aspiration present on x number of swallows<br>• Response to penetration<br>• Permanence of penetration<br>• Laryngeal vestibule residue volume<br>• Tracheal residue volume	**Epiglottic tilting**<br>*Rate when the body and / or distal tip of the epiglottis is in the position of maximal inversion.*<br>o 6: The body and distal tip of the epiglottis inverts completely and contacts the arytenoids.<br>o 5: The distal tip of epiglottis fails to completely invert and contact the arytenoids, although the arytenoid cartilages make contact with the body and base of epiglottis.<br>o 4: The body and distal tip of epiglottis fails to completely invert and contact the arytenoids, although the arytenoid cartilages make contact with the base of epiglottis.<br>o 3: The body of the epiglottis tilts substantially, but no part of the epiglottis (including the base) makes contact with the arytenoids.<br>o 2: Minimal body of epiglottis tilting and no part of the epiglottis makes contact with the arytenoids.<br>o 1: The body of the epiglottis does not appear to move from resting position and no part of the epiglottis makes contact with the arytenoids.
Penetration	• Penetration present on x number of swallows<br>• Penetration timing<br>• Penetration depth	**Penetration timing** **select all that apply*<br>o During swallow<br>o Before swallow initiated<br>o After swallow completed

Posterior Pharyngeal Function	• Pharyngeal wall movement<br>• Post. pharyngeal wall of hypopharynx residue volume<br>• Pyriform sinus residue volume<br>• Clearing Swallow Efficacy	**Pharyngeal wall movement**<br>*Top-down sequence of pharyngeal wall movement which follows the bolus tail. Review movement of pharyngeal wall between initiation of base of tongue posterior movement and upper oesopheageal sphincter opening of FIRST swallow.*<br>o 3: Posterior pharyngeal wall movement towards anterior pharynx is visible, with movement progressing from superior to inferior through the length of the pharynx<br>o 2: Anterior movement visible in one section of the pharyngeal wall only.<br>o 1: Nil movement visible.
Anterior-Posterior View	• Residue in Pyriform Sinuses – Volume<br>• Residue in Pyriform Sinuses – Symmetry<br>• Pharyngeal Wall Contraction – Symmetry<br>• Upper Oesophageal Sphincter Opening- Symmetry<br>• Upper Oesophageal Sphincter Opening- Width	**Residue in Pyriform Sinuses – Symmetry**<br>o 4: Minimal: a line coats the pyriform sinus; minimal residue is visible.<br>o 3: Mild: a small amount of material remains; residue is easily visible but contained within pyriform sinus (fills < half pyriform sinus space).<br>o 2: Moderate: Large volumes pool in the pyriform sinus, but do not fill or overflow the pyriform sinus (fills > half pyriform sinus space).<br>o 1: Substantial: Material is overflowing the pyriform sinus.

[a]*Full list is available upon request*
